# Crystal structure of the coordination polymer *catena*-poly[[bis­[hy­droxy(phen­yl)acetato-κ^2^ *O* ^1^,*O* ^2^]zinc(II)]-μ_2_-1,2-bis­(pyridin-4-yl)ethane-κ^2^ *N*:*N*′]

**DOI:** 10.1107/S2056989020014322

**Published:** 2020-11-24

**Authors:** Shen Fwu Ming, Lush Shie Fu

**Affiliations:** a Department of Medical Laboratory Science Biotechnology, Yuanpei University, No. 306, Yuanpei Street, Hsinchu, Taiwan 30015, ROC; b Department of Biotechnology, Yuanpei University, No. 306, Yuanpei Street, Hsinchu, Taiwan 30015, ROC

**Keywords:** crystal structure, coordination polymer, mandelate,1,2-bi(4-pyrid­yl)ethane, disorder

## Abstract

In the title polymeric Zn^II^ compound, [Zn(C_8_H_7_O_3_)_2_(C_12_H_12_N_2_)]_*n*_, the Zn cation is coordinated by two N atoms from 1,2-bis­(pyridin-4-yl)ethane unit and four O atoms from two mandelate [or hy­droxy(phen­yl)acetate] anions in a slightly distorted octa­hedral coordination geometry. The 1,2-bis­(pyridin-4-yl)ethane unit bridges two Zn^II^ cations, related by an inversion centre, to form a polymeric chain along [110]. The crystal structure features extensive O—H⋯O and weak C—H ⋯O hydrogen bonds, with C—H ⋯ π inter­actions and π–π inter­actions also being present. The centroid–centroid distance between the phenyl ring of the mandelate group and the 1,2-bis­(pyridine-4-yl)ethane moiety is 4.951 (2) Å. The 1,2-bis­(pyridin-4-yl)ethane ligand is disordered over two positions, with a refined occupancy of 0.578 (14) for the major component.

## Abstract 2

The synthesis and characterization of a new mandelato Zn^II^ complex with 1,2-bi(4-pyrid­yl)ethane is reported. The Zn cation is coordinated by two N atoms from the 1,2-bi(4-pyrid­yl)ethane unit and four O atoms from two mandelate anions in a slight distorted octa­hedral coordination geometry.

## Chemical context   

α-Hy­droxy­carb­oxy­lic acids play an important role in many biological processes and in coordination chemistry (Miyamoto *et al.*, 1989 ▸). The deprotonated anion of one example, mandelic acid (2-hy­droxy-2-phenyl­acetic acid), can behave as a multifunctional ligand and can act as a bridging ligand in metal complexes by involving the oxygen atoms of the carboxyl­ate and hy­droxy groups (Zechel *et al.*, 2019 ▸; Smatanová *et al.*, 2000 ▸; Bromant *et al.*, 2005 ▸). We report the preparation and structural characterization of a new coordin­ation polymer in which the Zn^II^ cations are coordin­ated to two mandelate anions, behaving as bidentate ligands, and linked together *via* 1,2-bis­(4-pyrid­yl)ethane mol­ecules. 1,2-Bis(4-pyrid­yl)ethane is a versatile building block for the purposes of crystal engineering as the pyridyl N atoms can connect to adjacent metals to form a chain (Lee & Kim, 2015 ▸). 
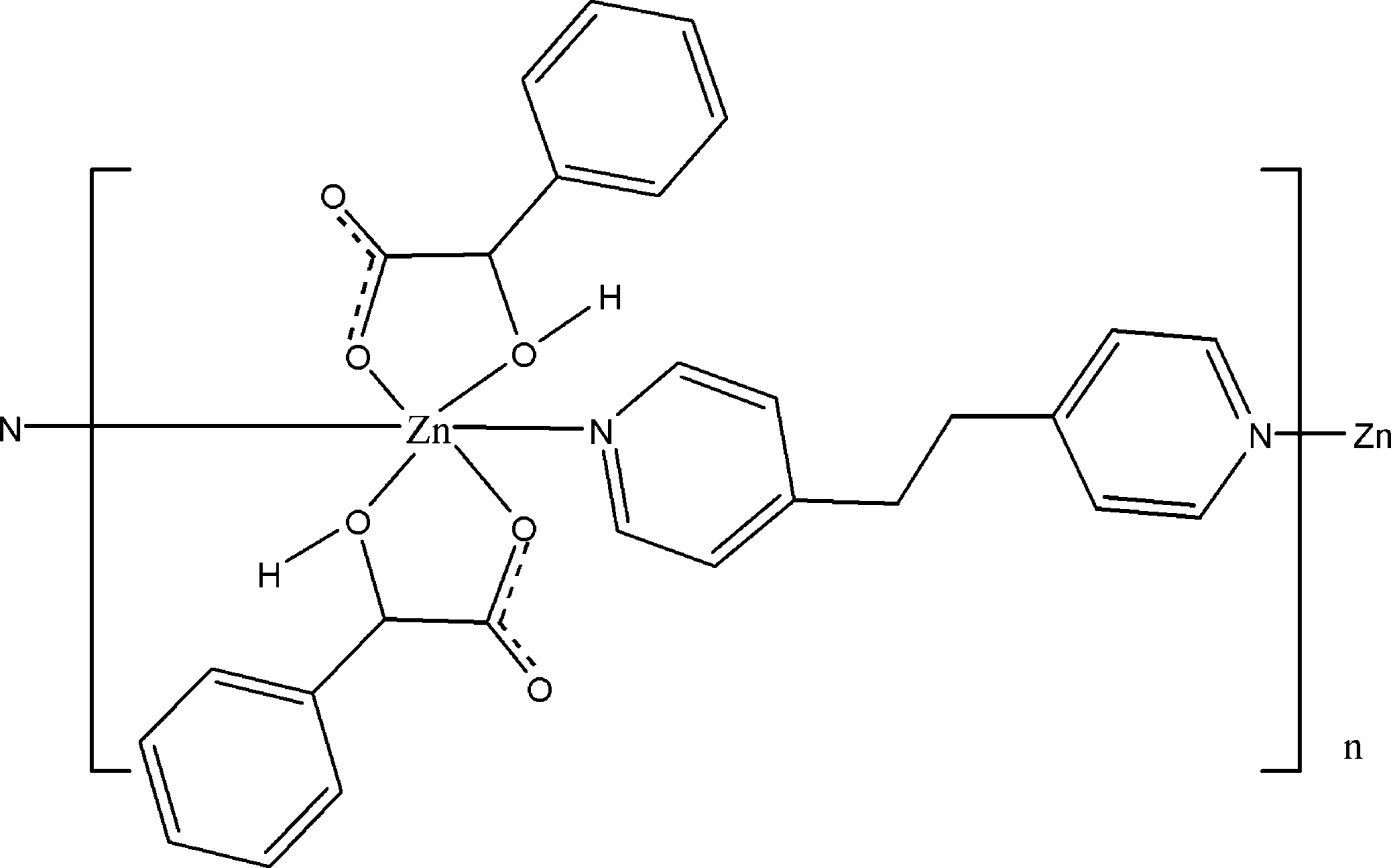


### Structural commentary   

**Figure 1:**
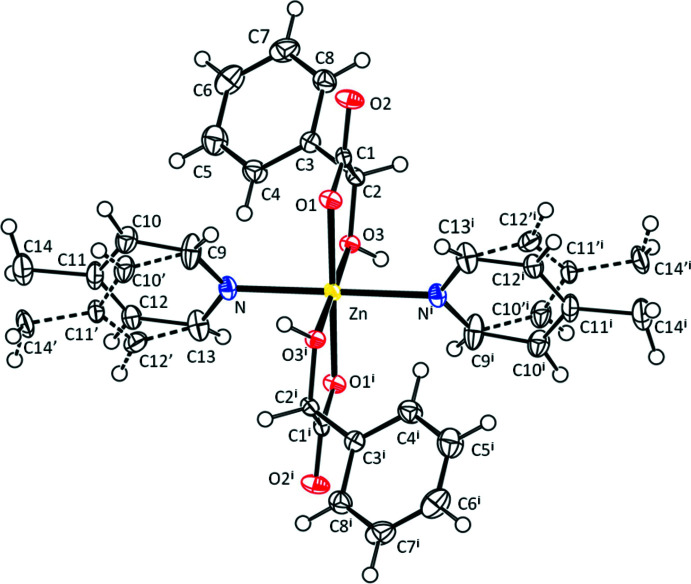
View of the title compound with the atom-numbering scheme. Displacement ellipsoids for non-H atoms are drawn at the 50% probability level [symmetry code: (i) −*x* + 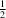, −*y* + 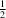, −*z*]. The major disorder component of the pyridine unit is drawn using solid lines and the minor disorder component is drawn using dashed lines.

The asymmetric unit of the title compound comprises one Zn^II^ cation, one mandelate anion and one half of a 1,2-bis­(4-pyridin-4-yl)ethane mol­ecule. There is an inversion centre located at the mid-point of the ethane C—C bond in the 1,2-bis­(4-pyridin-4-yl)ethane mol­ecule. Each Zn^II^ cation is coord­inated by two N atoms from two 1,2-bis­(4-pyridin-4-yl)ethane mol­ecules in a *trans* arrangement and four O atoms from two mandelate anions in a slightly distorted octa­hedral coordination geometry, as shown in Table 1 ▸ and Fig. 1 ▸. The mandelate anions are coordinated to the central Zn^2+^ cation form five-membered chelate rings *via* an oxygen atom of the OH group [Zn—O3 = 2.1013 (15) Å] and an oxygen atom of the carboxyl group [Zn—O1= 2.0290 (14) Å]. The Zn^II^ cations are linked together *via* 1,2-bis­(4-pyridin-4-yl)ethane bridges, forming a polymeric chain along [110].

## Supra­molecular features   

**Figure 2:**
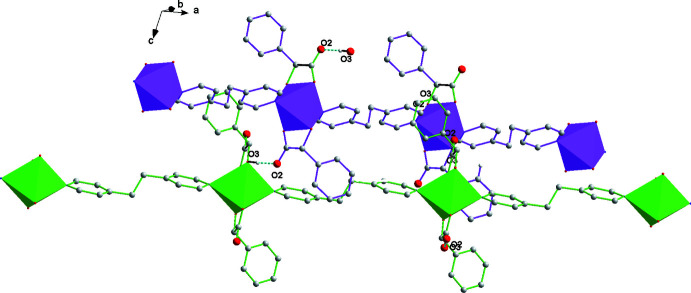
The mol­ecular packing of the title compound. Hydrogen bonds are shown as dashed lines. The minor occupancy components of the disordered pyridine carbon atoms have been omitted for clarity.

The crystal structure features extensive O—H⋯O hydrogen bonding [O3⋯O2^ii^ =2.572 (2) Å] (Fig. 2 ▸), establishing a three-dimensional network that is consolidated by further C—H⋯O hydrogen-bonding inter­actions. The C2—H2*A*⋯O1^ii^, C8—H8*A*⋯O2^iii^ and C13—H13*A*⋯O2^ii^ distances are 3.193 (2), 3.378 (3), and 3.064 (3) Å, respectively (Table 2 ▸). In addition, C—H ⋯π inter­actions [C9—H9*A*⋯*Cg*5^iv^ = 3.781 (2) Å and C12′–H12*B*⋯*Cg*5^ii^ = 3.649 (8) Å, Table 2 ▸] and π–π stacking are present in the crystal structure. The distance *Cg*5⋯*Cg*3^iv^ between the centroids of the phenyl ring (C3–C8) of the mandelate group and of the 1,2-bis­(pyridine-4-yl)ethane moiety (C9–C13) [symmetry code: (iv) −*x* + 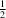, *y* + 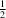, −*z* + 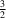] is 4.951 (2) Å and the dihedral angle between the two rings is 62.6 (2)°.

## Database survey   

Other examples of complexes containing the mandelate anion and the 1,2-bis­(pyridine-4-yl)ethane moiety were found in the Cambridge Structural Database (CSD, version 5.40, update of August 2019; Groom *et al.*, 2016 ▸). These include *catena*-[[μ-oxido(phen­yl)acetato](μ-4,4′-ethane-1,2-diyldi­pyridine)­zinc(II) perchlorate monohydrate] (CSD refcode QEBFUB; Guo *et al.*, 2015 ▸), which has a ClO_4_^−^ counter-ion. An Ni complex, *catena*-[bis­[(hy­droxy)(phen­yl)acetato]{μ-4-[2-(pyridin-4-yl)eth­yl]pyridine}­nickel(II)], isostructural with the title compound, has also been reported (QEBFAH; Guo *et al.*, 2015 ▸). A complex with the same mol­ecular formula but different coordination environment of the Zn atom, *catena*-[[μ_2_-1,2-bis­(4-pyrid­yl)ethane]­bis­(2-hy­droxy-2-phenyl­acetato)­zinc(II)] (MUBZEP; Yu *et al.*, 2009 ▸) has also been characterized. In this case, the 1,2-bis­(pyridine-4-yl)ethane and mandelate units are *cis* to each other.

## Synthesis and crystallization   

Zn(NO_3_)_2_ (91.4 mg, 0.50 mmol), 1,2-bi(4-pyrid­yl)ethane (92.1 mg, 0.50 mmol) and mandelic acid (76.0 mg, 0.50 mmol) were mixed in deionized water. The mixture was placed in a 25 mL Teflon linear reactor and heated at 423 K in an autoclave for 24 h. The resulting solution was slowly cooled to room temperature. Yellow transparent single crystals of the title compound were obtained in 75% yield (based on Zn).

## Refinement details   

Crystal data, data collection and structure refinement details are summarized in Table 3 ▸. Atoms C10, C11, C12, C14 of the pyridine ring are disordered over two sets of sites with an occupancy of 0.578 (14) for the major moiety. C-bound H atoms were included in calculated positions and treated as riding: C—H = 0.95 Å with *U*_iso_(H) = 1.5*U*_eq_(C-meth­yl) and 1.2*U*_eq_(C) for other H atoms·The hy­droxy H atoms, which could not be located in a difference-Fourier map, were included in idealized calculated positions that gave the most sensible geometry.

## Supplementary Material

Crystal structure: contains datablock(s) global, I. DOI: 10.1107/S2056989020014322/cq2039sup1.cif

Structure factors: contains datablock(s) I. DOI: 10.1107/S2056989020014322/cq2039Isup2.hkl

CCDC reference: 2040978

Additional supporting information:  crystallographic information; 3D view; checkCIF report

## Figures and Tables

**Table 1:** Selected geometric parameters (Å, °)

Zn—O1	2.0290 (14)	O2—C1	1.250 (3)
Zn—O3	2.1013 (15)	O3—C2	1.424 (2)
Zn—N	2.2217 (19)	N—C13	1.334 (3)
O1—C1	1.260 (2)	N—C9	1.339 (3)
			
O1—Zn—O3	80.02 (6)	C9—N—C13	117.3 (2)
O1—Zn—N	90.25 (7)	O2—C1—C2	116.10 (17)
O1—Zn—O3^i^	99.98 (6)	O1—C1—O2	124.67 (19)
O1—Zn—N^i^	89.75 (7)	O1—C1—C2	119.21 (18)
O3—Zn—N	88.73 (6)	O3—C2—C3	110.90 (18)
O3—Zn—N^i^	91.27 (6)	O3—C2—C1	110.17 (16)
Zn—O1—C1	116.96 (13)	N—C9—C10	118.8 (4)
Zn—O3—C2	113.41 (12)	N—C9—C10′	127.1 (6)
Zn—N—C13	120.03 (16)	N—C13—C12′	116.3 (4)
Zn—N—C9	122.45 (16)	N—C13—C12	126.5 (3)

Symmetry code: (i) 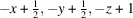.

**Table 2:** Hydrogen-bond geometry (Å, °) *Cg*5 is the centroid of the C3–C8 ring.

*D*—H⋯*A*	*D*—H	H⋯*A*	*D*⋯*A*	*D*—H⋯*A*
O3—H3*A*⋯O2^ii^	0.86 (3)	1.72 (3)	2.572 (2)	177.3 (15)
C2—H2*A*⋯O1^ii^	1.00	2.46	3.193 (2)	129
C8—H8*A*⋯O2^iii^	0.95	2.44	3.378 (3)	168
C13—H13*A*⋯O2^ii^	0.96	2.43	3.064 (3)	124
C9—H9*A*⋯*Cg*5^iv^	0.96	2.88	3.781 (2)	157
C12′—H12*B*⋯*Cg*5^ii^	0.95	2.75	3.649 (8)	159

Symmetry codes: (ii) 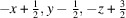; (iii) 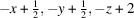; (iv) 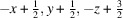.

**Table 3:** Experimental details

Crystal data	
Chemical formula	[Zn(C_8_H_7_O_3_)_2_(C_12_H_12_N_2_)]
*M*_r_	551.90
Crystal system, space group	Monoclinic, *C*2/*c*
Temperature (K)	150
*a*, *b*, *c* (Å)	25.6754 (19), 9.8838 (5), 10.6208 (7)
β (°)	108.234 (7)
*V* (Å^3^)	2559.9 (3)
*Z*	4
Radiation type	Mo *K*α
μ (mm^−1^)	1.01
Crystal size (mm)	0.35 × 0.32 × 0.26

Data collection	
Diffractometer	Oxford Diffraction Gemini-S CCD detector
Absorption correction	Multi-scan (*CrysAlis PRO*; Oxford Diffraction, 2009 ▸)
*T*_min_, *T*_max_	0.936, 1.000
No. of measured, independent and observed [*I* > 2σ(*I*)] reflections	5016, 2270, 1970
*R*_int_	0.027

Refinement	
*R*[*F* ^2^ > 2σ(*F* ^2^)], *wR*(*F* ^2^), *S*	0.031, 0.082, 1.04
No. of reflections	2270
No. of parameters	210
H-atom treatment	H atoms treated by a mixture of independent and constrained refinement
Δρ_max_, Δρ_min_ (e Å^−3^)	0.43, −0.41

Computer programs: *CrysAlis PRO* (Oxford Diffraction, 2009 ▸), *SHELXS97* and *SHELXL97* (Sheldrick, 2008 ▸), *DIAMOND* (Brandenburg & Putz, 1999 ▸) and *PLATON* (Spek, 2020 ▸).

## supplementary crystallographic information

### Crystal data

**Table:**

[Zn(C_8_H_7_O_3_)_2_(C_12_H_12_N_2_)]	*F*(000) = 1144
*M**_r_* = 551.90	*D*_x_ = 1.432 Mg m^−^^3^
Monoclinic, *C*2/*c*	Mo *K*α radiation, λ = 0.71073 Å
Hall symbol: -C 2yc	Cell parameters from 1818 reflections
*a* = 25.6754 (19) Å	θ = 2.8–29.2°
*b* = 9.8838 (5) Å	µ = 1.00 mm^−^^1^
*c* = 10.6208 (7) Å	*T* = 150 K
β = 108.234 (7)°	Parallelepiped, yellow
*V* = 2559.9 (3) Å^3^	0.35 × 0.32 × 0.26 mm
*Z* = 4	

### Data collection

**Table:**

Oxford Diffraction Gemini-S CCD detector diffractometer	2270 independent reflections
Radiation source: fine-focus sealed tube	1970 reflections with *I* > 2σ(*I*)
Graphite monochromator	*R*_int_ = 0.027
ω scans	θ_max_ = 25.0°, θ_min_ = 2.8°
Absorption correction: multi-scan (CrysAlisPro; Oxford Diffraction, 2009)	*h* = −23→30
*T*_min_ = 0.936, *T*_max_ = 1.000	*k* = −7→11
5016 measured reflections	*l* = −12→11

### Refinement

**Table:**

Refinement on *F*^2^	Primary atom site location: structure-invariant direct methods
Least-squares matrix: full	Secondary atom site location: difference Fourier map
*R*[*F*^2^ > 2σ(*F*^2^)] = 0.031	Hydrogen site location: inferred from neighbouring sites
*wR*(*F*^2^) = 0.082	H atoms treated by a mixture of independent and constrained refinement
*S* = 1.04	*w* = 1/[σ^2^(*F*_o_^2^) + (0.038*P*)^2^ + 2.1396*P*] where *P* = (*F*_o_^2^ + 2*F*_c_^2^)/3
2270 reflections	(Δ/σ)_max_ < 0.001
210 parameters	Δρ_max_ = 0.43 e Å^−^^3^
0 restraints	Δρ_min_ = −0.40 e Å^−^^3^

### Special details

**Table:**

Geometry. Bond distances, angles etc. have been calculated using the rounded fractional coordinates. All su's are estimated from the variances of the (full) variance-covariance matrix. The cell esds are taken into account in the estimation of distances, angles and torsion angles

### Fractional atomic coordinates and isotropic or equivalent isotropic displacement parameters (Å^2^)

**Table:**

	*x*	*y*	*z*	*U*_iso_*/*U*_eq_	Occ. (<1)
Zn	0.25000	0.25000	0.50000	0.0138 (1)	
O1	0.25093 (6)	0.37191 (14)	0.65439 (14)	0.0171 (5)	
O2	0.26248 (7)	0.37664 (14)	0.87065 (15)	0.0229 (5)	
O3	0.28062 (6)	0.11415 (15)	0.65814 (14)	0.0151 (5)	
N	0.16575 (7)	0.18198 (19)	0.48454 (18)	0.0189 (6)	
C1	0.26431 (9)	0.3184 (2)	0.7677 (2)	0.0146 (6)	
C2	0.28282 (9)	0.1695 (2)	0.7835 (2)	0.0154 (7)	
C3	0.33972 (9)	0.1537 (2)	0.8826 (2)	0.0164 (6)	
C4	0.38568 (10)	0.1438 (2)	0.8401 (2)	0.0249 (7)	
C5	0.43745 (11)	0.1262 (3)	0.9311 (3)	0.0349 (9)	
C6	0.44387 (11)	0.1178 (3)	1.0644 (3)	0.0352 (9)	
C7	0.39842 (11)	0.1278 (2)	1.1077 (2)	0.0319 (8)	
C8	0.34670 (10)	0.1461 (2)	1.0174 (2)	0.0235 (7)	
C9	0.13113 (11)	0.2581 (2)	0.5259 (3)	0.0279 (8)	
C10	0.0768 (3)	0.2104 (9)	0.5048 (9)	0.0261 (19)	0.578 (14)
C11	0.0594 (2)	0.0835 (5)	0.4523 (9)	0.026 (2)	0.578 (14)
C12	0.0972 (3)	0.0046 (6)	0.4172 (10)	0.0260 (18)	0.578 (14)
C13	0.14957 (10)	0.0569 (2)	0.4432 (3)	0.0310 (8)	
C14	0.0017 (3)	0.0308 (7)	0.4359 (5)	0.0341 (17)	0.578 (14)
C12'	0.1046 (4)	0.0024 (8)	0.4827 (13)	0.025 (3)	0.422 (14)
C14'	0.0228 (4)	0.0209 (9)	0.5626 (7)	0.028 (3)	0.422 (14)
C10'	0.0870 (5)	0.2148 (13)	0.5599 (12)	0.026 (3)	0.422 (14)
C11'	0.0722 (3)	0.0805 (6)	0.5343 (12)	0.021 (2)	0.422 (14)
H4A	0.38160	0.14900	0.74820	0.0300*	
H7A	0.40270	0.12210	1.19970	0.0380*	
H5A	0.46860	0.12000	0.90120	0.0420*	
H6A	0.47930	0.10510	1.12650	0.0420*	
H10A	0.05140	0.26770	0.52760	0.0320*	0.578 (14)
H12A	0.08770	−0.08160	0.37700	0.0310*	0.578 (14)
H13A	0.17770	−0.00500	0.43840	0.0370*	
H14A	−0.02490	0.10630	0.40920	0.0410*	0.578 (14)
H14B	−0.00830	−0.03820	0.36490	0.0410*	0.578 (14)
H8A	0.31580	0.15360	1.04800	0.0280*	
H9A	0.13500	0.35420	0.51880	0.0330*	
H2A	0.25660	0.11780	0.81760	0.0180*	
H3A	0.2653 (11)	0.036 (3)	0.647 (3)	0.039 (8)*	
H10B	0.06730	0.27390	0.59920	0.0310*	0.422 (14)
H12B	0.09720	−0.09190	0.47220	0.0300*	0.422 (14)
H14C	0.00800	0.08830	0.61150	0.0340*	0.422 (14)
H14D	0.03450	−0.05930	0.62030	0.0340*	0.422 (14)

### Atomic displacement parameters (Å^2^)

**Table:**

	*U*^11^	*U*^22^	*U*^33^	*U*^12^	*U*^13^	*U*^23^
Zn	0.0156 (2)	0.0132 (2)	0.0143 (2)	−0.0016 (2)	0.0070 (2)	−0.0005 (1)
O1	0.0242 (9)	0.0122 (7)	0.0164 (8)	0.0026 (7)	0.0086 (6)	0.0012 (6)
O2	0.0352 (10)	0.0182 (8)	0.0176 (8)	0.0074 (7)	0.0115 (7)	−0.0001 (6)
O3	0.0222 (9)	0.0085 (7)	0.0157 (8)	−0.0012 (7)	0.0075 (6)	−0.0007 (6)
N	0.0166 (10)	0.0191 (10)	0.0217 (10)	−0.0016 (9)	0.0072 (8)	0.0046 (8)
C1	0.0134 (11)	0.0133 (11)	0.0193 (11)	−0.0012 (9)	0.0084 (9)	−0.0006 (9)
C2	0.0208 (12)	0.0109 (11)	0.0183 (11)	−0.0013 (10)	0.0115 (9)	−0.0014 (8)
C3	0.0214 (12)	0.0088 (10)	0.0196 (11)	0.0000 (9)	0.0074 (9)	0.0015 (8)
C4	0.0221 (13)	0.0303 (13)	0.0227 (12)	0.0043 (11)	0.0075 (10)	0.0005 (10)
C5	0.0222 (14)	0.0431 (16)	0.0388 (16)	0.0047 (13)	0.0089 (12)	0.0005 (12)
C6	0.0274 (15)	0.0358 (15)	0.0324 (15)	0.0032 (12)	−0.0049 (12)	0.0040 (12)
C7	0.0412 (17)	0.0305 (14)	0.0187 (12)	−0.0026 (13)	0.0016 (11)	0.0041 (10)
C8	0.0298 (14)	0.0198 (12)	0.0234 (12)	−0.0020 (11)	0.0118 (10)	0.0015 (10)
C9	0.0229 (14)	0.0229 (13)	0.0414 (15)	0.0022 (11)	0.0151 (12)	0.0065 (11)
C10	0.014 (3)	0.038 (3)	0.028 (4)	−0.001 (3)	0.009 (3)	−0.005 (4)
C11	0.016 (2)	0.032 (3)	0.030 (5)	−0.007 (2)	0.007 (3)	0.005 (2)
C12	0.024 (3)	0.020 (2)	0.034 (4)	−0.003 (2)	0.009 (3)	−0.001 (3)
C13	0.0174 (13)	0.0188 (13)	0.0563 (17)	0.0011 (11)	0.0110 (12)	0.0067 (12)
C14	0.022 (3)	0.042 (3)	0.040 (3)	−0.006 (3)	0.012 (2)	0.007 (3)
C12'	0.024 (4)	0.016 (3)	0.036 (6)	−0.005 (3)	0.011 (4)	−0.002 (4)
C14'	0.026 (5)	0.031 (4)	0.035 (4)	−0.008 (4)	0.020 (3)	0.002 (3)
C10'	0.022 (5)	0.029 (4)	0.025 (6)	−0.004 (3)	0.006 (5)	−0.011 (5)
C11'	0.014 (3)	0.028 (3)	0.021 (6)	−0.005 (3)	0.007 (3)	0.001 (3)

### Geometric parameters (Å, º)

**Table:**

Zn—O1	2.0290 (14)	C11—C12	1.384 (10)
Zn—O3	2.1013 (15)	C11—C14	1.528 (10)
Zn—N	2.2217 (19)	C11'—C14'	1.512 (13)
Zn—O1^i^	2.0290 (14)	C11'—C12'	1.369 (14)
Zn—O3^i^	2.1013 (15)	C12—C13	1.385 (9)
Zn—N^i^	2.2217 (19)	C12'—C13	1.450 (11)
O1—C1	1.260 (2)	C14—C14^ii^	1.519 (8)
O2—C1	1.250 (3)	C14'—C14'^ii^	1.528 (12)
O3—C2	1.424 (2)	C2—H2A	1.0000
O3—H3A	0.86 (3)	C4—H4A	0.9500
N—C13	1.334 (3)	C5—H5A	0.9500
N—C9	1.339 (3)	C6—H6A	0.9500
C1—C2	1.540 (3)	C7—H7A	0.9500
C2—C3	1.518 (3)	C8—H8A	0.9500
C3—C8	1.388 (3)	C9—H9A	0.9600
C3—C4	1.393 (4)	C10—H10A	0.9500
C4—C5	1.388 (4)	C10'—H10B	0.9500
C5—C6	1.376 (4)	C12—H12A	0.9500
C6—C7	1.385 (4)	C12'—H12B	0.9500
C7—C8	1.385 (3)	C13—H13A	0.9600
C9—C10	1.422 (9)	C14—H14B	0.9900
C9—C10'	1.361 (14)	C14—H14A	0.9900
C10—C11	1.388 (11)	C14'—H14C	0.9900
C10'—C11'	1.384 (14)	C14'—H14D	0.9900
			
O1···O3	2.656 (2)	C9···H4A^i^	2.9700
O1···N	3.015 (2)	C10···H14B^ii^	3.0700
O1···C2	2.419 (2)	C10'···H7A^iv^	2.9600
O1···C9	3.156 (3)	C12'···H14C^ii^	2.8900
O1···C13^i^	3.123 (3)	C13···H3A	3.09 (3)
O1···C2^iii^	3.193 (2)	C14'···H12B^ii^	3.0700
O1···N^i^	3.002 (2)	H2A···O1^v^	2.4600
O1···O3^i^	3.164 (2)	H2A···C1^v^	3.0900
O2···C8^iv^	3.378 (3)	H2A···H8A	2.4700
O2···O3^iii^	2.572 (2)	H3A···O1^v^	2.79 (3)
O2···C2^iii^	3.350 (2)	H3A···C13	3.09 (3)
O2···C8	3.192 (3)	H3A···O2^v^	1.72 (3)
O2···C13^iii^	3.064 (3)	H3A···C1^v^	2.54 (3)
O3···O1^i^	3.164 (2)	H4A···O3	2.4900
O3···N	3.024 (2)	H4A···N^i^	2.9200
O3···C1	2.431 (3)	H4A···C9^i^	2.9700
O3···O2^v^	2.572 (2)	H5A···H10A^vi^	2.4000
O3···C1^v^	3.326 (3)	H6A···H6A^vii^	2.5000
O3···O1	2.656 (2)	H7A···C10'^iv^	2.9600
O3···N^i^	3.092 (2)	H7A···H10B^iv^	2.2800
O1···H13A^i^	2.6800	H8A···H2A	2.4700
O1···H3A^iii^	2.79 (3)	H8A···O2^iv^	2.4400
O1···H9A	2.8800	H9A···C8^iii^	2.9700
O1···H2A^iii^	2.4600	H9A···C7^iii^	3.0200
O2···H8A^iv^	2.4400	H9A···O1	2.8800
O2···H3A^iii^	1.72 (3)	H10A···H14A	2.5300
O2···H13A^iii^	2.4300	H10A···C5^viii^	2.9700
O3···H4A	2.4900	H10A···H5A^viii^	2.4000
N···O1	3.015 (2)	H10B···H14C	2.4100
N···O3	3.024 (2)	H10B···H7A^iv^	2.2800
N···O1^i^	3.002 (2)	H12A···H14B	2.4700
N···O3^i^	3.092 (2)	H12A···C7^v^	2.8900
N···H4A^i^	2.9200	H12B···H14D	2.6000
C1···O3^iii^	3.326 (3)	H12B···C14'^ii^	3.0700
C2···O2^v^	3.350 (2)	H12B···C7^v^	2.9100
C2···O1^v^	3.193 (2)	H12B···C8^v^	2.9500
C8···O2	3.192 (3)	H12B···H14C^ii^	2.5700
C8···O2^iv^	3.378 (3)	H12B···C6^v^	3.0400
C13···O2^v^	3.064 (3)	H13A···O1^i^	2.6800
C1···H3A^iii^	2.54 (3)	H13A···O2^v^	2.4300
C1···H2A^iii^	3.0900	H14A···C5^viii^	2.8500
C5···H10A^vi^	2.9700	H14A···H10A	2.5300
C5···H14A^vi^	2.8500	H14B···H12A	2.4700
C6···H12B^iii^	3.0400	H14B···C10^ii^	3.0700
C7···H12B^iii^	2.9100	H14C···H10B	2.4100
C7···H12A^iii^	2.8900	H14C···C12'^ii^	2.8900
C7···H9A^v^	3.0200	H14C···H12B^ii^	2.5700
C8···H12B^iii^	2.9500	H14D···H12B	2.6000
C8···H9A^v^	2.9700		
			
O1—Zn—O3	80.02 (6)	C10'—C11'—C14'	122.1 (9)
O1—Zn—N	90.25 (7)	C11—C12—C13	117.1 (6)
O1—Zn—O1^i^	180.00	C11'—C12'—C13	123.0 (7)
O1—Zn—O3^i^	99.98 (6)	N—C13—C12'	116.3 (4)
O1—Zn—N^i^	89.75 (7)	N—C13—C12	126.5 (3)
O3—Zn—N	88.73 (6)	C11—C14—C14^ii^	111.1 (6)
O1^i^—Zn—O3	99.98 (6)	C11'—C14'—C14'^ii^	113.2 (7)
O3—Zn—O3^i^	180.00	O3—C2—H2A	108.00
O3—Zn—N^i^	91.27 (6)	C1—C2—H2A	108.00
O1^i^—Zn—N	89.75 (7)	C3—C2—H2A	108.00
O3^i^—Zn—N	91.27 (6)	C3—C4—H4A	120.00
N—Zn—N^i^	180.00	C5—C4—H4A	120.00
O1^i^—Zn—O3^i^	80.02 (6)	C4—C5—H5A	120.00
O1^i^—Zn—N^i^	90.25 (7)	C6—C5—H5A	120.00
O3^i^—Zn—N^i^	88.73 (6)	C5—C6—H6A	120.00
Zn—O1—C1	116.96 (13)	C7—C6—H6A	120.00
Zn—O3—C2	113.41 (12)	C6—C7—H7A	120.00
C2—O3—H3A	111 (2)	C8—C7—H7A	120.00
Zn—O3—H3A	115 (2)	C3—C8—H8A	120.00
Zn—N—C13	120.03 (16)	C7—C8—H8A	120.00
Zn—N—C9	122.45 (16)	N—C9—H9A	116.00
C9—N—C13	117.3 (2)	C10—C9—H9A	116.00
O2—C1—C2	116.10 (17)	C10'—C9—H9A	117.00
O1—C1—O2	124.67 (19)	C9—C10—H10A	119.00
O1—C1—C2	119.21 (18)	C11—C10—H10A	119.00
O3—C2—C3	110.90 (18)	C9—C10'—H10B	122.00
O3—C2—C1	110.17 (16)	C11'—C10'—H10B	122.00
C1—C2—C3	111.79 (17)	C11—C12—H12A	122.00
C2—C3—C8	120.4 (2)	C13—C12—H12A	121.00
C2—C3—C4	120.78 (18)	C13—C12'—H12B	119.00
C4—C3—C8	118.8 (2)	C11'—C12'—H12B	118.00
C3—C4—C5	120.5 (2)	N—C13—H13A	116.00
C4—C5—C6	120.3 (3)	C12—C13—H13A	117.00
C5—C6—C7	119.7 (3)	C12'—C13—H13A	117.00
C6—C7—C8	120.3 (2)	C14^ii^—C14—H14B	109.00
C3—C8—C7	120.5 (2)	H14A—C14—H14B	108.00
N—C9—C10	118.8 (4)	C14^ii^—C14—H14A	109.00
N—C9—C10'	127.1 (6)	C11—C14—H14A	109.00
C9—C10—C11	122.6 (7)	C11—C14—H14B	109.00
C9—C10'—C11'	116.7 (10)	C11'—C14'—H14C	109.00
C12—C11—C14	120.9 (6)	C11'—C14'—H14D	109.00
C10—C11—C14	122.2 (6)	H14C—C14'—H14D	108.00
C10—C11—C12	116.9 (6)	C14'^ii^—C14'—H14C	109.00
C12'—C11'—C14'	120.9 (7)	C14'^ii^—C14'—H14D	109.00
C10'—C11'—C12'	117.1 (9)		
			
O3—Zn—O1—C1	−4.48 (16)	O1—C1—C2—C3	122.9 (2)
N—Zn—O1—C1	84.19 (17)	O2—C1—C2—O3	177.3 (2)
O3^i^—Zn—O1—C1	175.52 (16)	O2—C1—C2—C3	−58.9 (3)
N^i^—Zn—O1—C1	−95.81 (17)	O3—C2—C3—C4	25.8 (3)
O1—Zn—O3—C2	3.79 (14)	O3—C2—C3—C8	−152.94 (18)
N—Zn—O3—C2	−86.69 (14)	C1—C2—C3—C4	−97.6 (2)
O1^i^—Zn—O3—C2	−176.21 (14)	C1—C2—C3—C8	83.7 (2)
N^i^—Zn—O3—C2	93.31 (14)	C2—C3—C4—C5	−178.5 (2)
O1—Zn—N—C9	25.77 (19)	C8—C3—C4—C5	0.2 (3)
O1—Zn—N—C13	−148.46 (19)	C2—C3—C8—C7	178.15 (18)
O3—Zn—N—C9	105.78 (19)	C4—C3—C8—C7	−0.6 (3)
O3—Zn—N—C13	−68.45 (19)	C3—C4—C5—C6	0.3 (4)
O1^i^—Zn—N—C9	−154.23 (19)	C4—C5—C6—C7	−0.4 (4)
O1^i^—Zn—N—C13	31.54 (19)	C5—C6—C7—C8	0.0 (4)
O3^i^—Zn—N—C9	−74.22 (19)	C6—C7—C8—C3	0.5 (3)
O3^i^—Zn—N—C13	111.55 (19)	N—C9—C10—C11	5.1 (10)
Zn—O1—C1—O2	−173.77 (19)	C9—C10—C11—C12	−1.7 (13)
Zn—O1—C1—C2	4.3 (3)	C9—C10—C11—C14	177.2 (6)
Zn—O3—C2—C1	−2.7 (2)	C10—C11—C12—C13	2.9 (12)
Zn—O3—C2—C3	−127.04 (14)	C14—C11—C12—C13	−176.0 (6)
Zn—N—C9—C10	176.2 (4)	C10—C11—C14—C14^ii^	−83.6 (9)
C13—N—C9—C10	−9.5 (5)	C12—C11—C14—C14^ii^	95.2 (9)
Zn—N—C13—C12	−173.7 (5)	C11—C12—C13—N	−8.3 (11)
C9—N—C13—C12	11.8 (6)	C11—C14—C14^ii^—C11^ii^	180.0 (5)
O1—C1—C2—O3	−0.9 (3)		

Symmetry codes: (i) −*x*+1/2, −*y*+1/2, −*z*+1; (ii) −*x*, −*y*, −*z*+1; (iii) −*x*+1/2, *y*+1/2, −*z*+3/2; (iv) −*x*+1/2, −*y*+1/2, −*z*+2; (v) −*x*+1/2, *y*−1/2, −*z*+3/2; (vi) *x*+1/2, −*y*+1/2, *z*+1/2; (vii) −*x*+1, *y*, −*z*+5/2; (viii) *x*−1/2, −*y*+1/2, *z*−1/2.

### Hydrogen-bond geometry (Å, º)

*Cg*5 is the centroid of the C3–C8 ring.

**Table:**

*D*—H···*A*	*D*—H	H···*A*	*D*···*A*	*D*—H···*A*
O3—H3*A*···O2^v^	0.86 (3)	1.72 (3)	2.572 (2)	177.3 (15)
C2—H2*A*···O1^v^	1.00	2.46	3.193 (2)	129
C8—H8*A*···O2^iv^	0.95	2.44	3.378 (3)	168
C13—H13*A*···O2^v^	0.96	2.43	3.064 (3)	124
C9—H9*A*···*Cg*5^iii^	0.96	2.88	3.781 (2)	157
C12′—H12*B*···*Cg*5^v^	0.95	2.75	3.649 (8)	159

Symmetry codes: (iii) −*x*+1/2, *y*+1/2, −*z*+3/2; (iv) −*x*+1/2, −*y*+1/2, −*z*+2; (v) −*x*+1/2, *y*−1/2, −*z*+3/2.
